# Nonsubjective Assessment of Shape, Volume and Symmetry during Breast Augmentation with Handheld 3D Device

**DOI:** 10.3390/jcm11144002

**Published:** 2022-07-11

**Authors:** Zhouxiao Li, Thilo Ludwig Schenck, Riccardo Enzo Giunta, Lucas Etzel, Konstantin Christoph Koban

**Affiliations:** Division of Hand, Plastic and Aesthetic Surgery, University Hospital, LMU Munich, 80336 Munich, Germany; schenck@geisenhoferklinik.de (T.L.S.); riccardo.giunta@med.uni-muenchen.de (R.E.G.); lucas.etzel@med.uni-muenchen.de (L.E.); konstantin.koban@med.uni-muenchen.de (K.C.K.)

**Keywords:** breast augmentation, breast asymmetry, 3D surface imaging, intraoperative measurement, volume and surface difference

## Abstract

Three-dimensional Surface Imaging (3DSI) has become a valuable tool for planning and documenting surgical procedures. Although surface scanners have allowed for a better understanding of breast shape, size, and asymmetry during patient consultation, its use has not been included in intraoperative assessment so far. Validation of the reliability of the intraoperative use of a portable handheld 3DSI equipment as a tool to evaluate morphological changes during breast augmentation surgery. The patients who underwent bilateral subpectoral breast augmentation through an inframammary incision were included in this study. Intraoperative 3DSI was performed with the Artec Eva device, allowing for visualization of the surgical area before incision, after use of breast sizers and implant, and after wound closure. Intraoperatively manual measurements of breast distances and volume changes due to known sizer and implant volumes were in comparison with digital measurements calculated from 3DSI of the surgical area. Bilateral breasts of 40 patients were 3D photographed before incision and after suture successfully. A further 108 implant sizer uses were digitally documented. There was no significant difference between manual tape measurement and digital breast distance measurement. Pre- to postoperative 3D volume change showed no significant difference to the known sizer and implant volume.

## 1. Introduction

Breast augmentation continues to be the most demanded and performed aesthetic plastic surgery since 2006, with more than 315,000 annual procedures in the US [1]. Although there does not seem to be a universal “perfect” breast [2], choosing the right implant size and shape can be challenging, as up to 40–44% of patients present asymmetric shape of breast and up to 53% an asymmetry in size [3,4].

Three-dimensional Surface Imaging (3DSI) has proven its worth in preoperative consultation and postoperative documentation of breast surgery with known advantages and disadvantages, such as non-contact digital acquisition of breast parameters, breast volumes, symmetry values and preoperative simulation of breast augmentation, but also the limitations of pronounced breast hypertrophy, short inter-breast distance and ptosis with incorrect interpolation of breast volumes and breast parameters [5,6,7,8,9,10,11]. Studies showed a high interest and positive experiences of the patients [6,12,13,14]. Through the development of various mobile handheld 3D scanners [15,16], in addition to the pure documentation and planning of plastic surgery, the intraoperative application as in the augmented-reality evaluation of facial surgery [17] and in breast surgery and radiation treatment [18,19,20] has been described based on case reports.

Choosing the appropriate volume and shape of breast implant is essential for the desirable and natural postoperative breast appearance and reducing postoperative complications such as contracture and rupture of the implant. Currently, the choice of the implant is still mainly based on the patients’ subjective desire and the surgeons’ manual tape measurement before the operation [21]. However, surgeons often find that the effect of the selected implant is not as satisfactory as that of preoperative evaluation [22,23]. Moreover, sometimes multiple candidate breast implants can also produce controversial aesthetic evaluations, troubling the surgeon in making the appropriate decision [24]. We believe that plastic surgeons pick out more suitable implants with intraoperatively gathered objective data acquired using 3DSI of the breast.

The main purpose of the study was to validate the intraoperative application of a movable handheld 3D surface imaging device as an objective instrument for morphological changes evaluation during breast augmentation process. Therefore, intraoperatively gauged manual breast distances as well as volume variations due to implant and foregone sizer were compared with digital measurements computed from 3D surface images of the operation area.

In addition, we evaluated 3D surfaces and volumetric symmetry for investigating the question of when and to what extent intraoperative 3DSI might be helpful in the future.

## 2. Patients, Materials and Methods

### 2.1. Study Population

The enrolled samples were patients who planned for bilateral, submuscular breast augmentation with silicone implants through an inframammary incision/method, for a previous study could show breast parenchymal atrophy by MRI due to sub-glandular implant placement, which could potentially limit the 3D measurement of breast volume changes in these particular cases [25]. Exclusion criteria are being under 18 years of age, having a history of breast lesion, breast surgery or breast cancer, having congenital breast deformities, significant shape abnormalities or ptosis, scoliosis, or chest wall deformities, or if they were to undergo additional breast plastic surgery such as mastopexy or fat grafting.

### 2.2. Surgical Consultation and Implant Size

Each breast augmentation was conducted by either a chief or senior consultant. All study participants received a routine preoperative patient consultation, during which 28 of the subjects chose between two implants of the same shape but different volume. In 12 cases where subjects presented themselves with significant breast volume and surface asymmetry, intraoperative determination of the appropriate implant shape and size was planned. For this purpose, the relevant subjects selected a minimum and maximum implant volume. The final implants were selected based on optimal breast symmetry, shape and size according to the patient’s wishes and the surgeon’s subjective opinion. All patients went with Mentor (Irvine, CA, USA) silicon sizers and implants.

### 2.3. Intraoperative 3D Surface Imaging

Corresponding with the surgeon’s view of the operating field, 3D surface images were obtained using the structured-light scanner Artec Eva (Artec 3D, Luxembourg) mounted on a handheld monopod for ease of use [26,27]. Three-dimensional Surface Imaging was performed at the beginning of surgery before the first incision, after each sizer implantation or exchange, and at the end of surgery after wound closure with the final implant in place. There was no alteration regarding standardized breast surgical routine.

**Figure 1:**
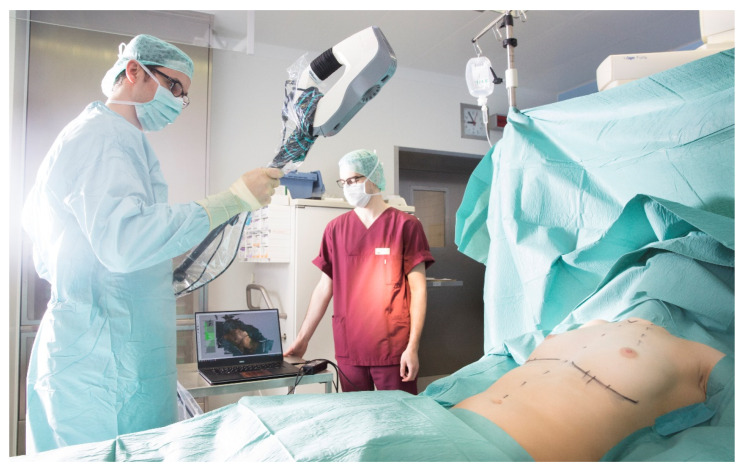
The intraoperative scan with 3DSI for a patient who underwent submuscular breast augmentation.

Each imaging procedure was conducted while the subject’s torso was seated upright at an angle of approximately 90 degrees on the same adjustable operating table. Preliminary examinations showed minimal subject movement when changing between supine and upright position. The operating field was systematically imaged using a zigzag pattern, with the scanner held at an operating distance of approximately 90 cm. Three-dimensional reconstruction of the gathered point cloud was performed using Artec Studio 12 (Artec 3D, Luxembourg) in high resolution setting. Images were then exported and evaluated (Figure 1)using the Mirror medical imaging software (Canfield Scientific Inc., Parsippany-Troy Hills, NJ, USA) [6,14].

**Figure 2:**
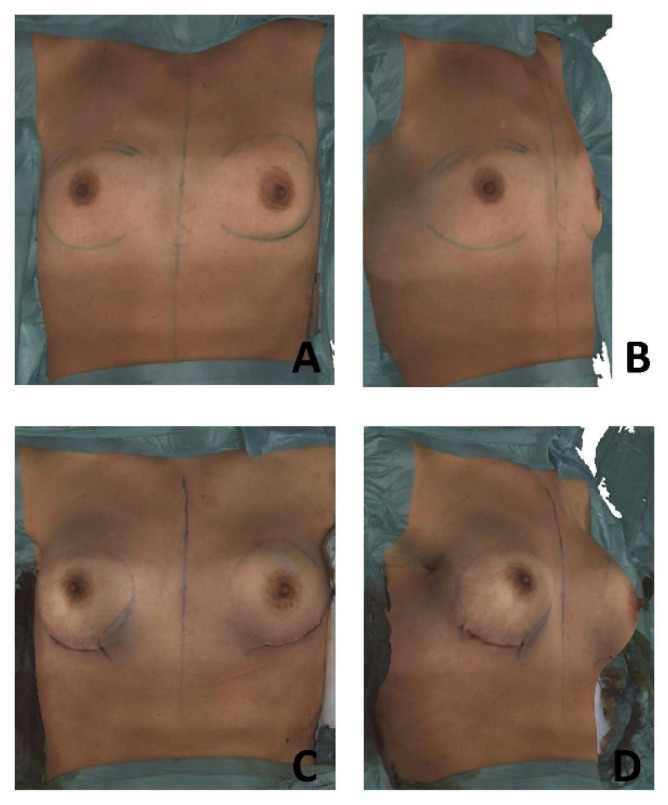
Preoperative 3D Surface Image in operating room (O.R., frontal (**A**); oblique view (**B**)) and postoperative 3D Surface Image in O.R. after implant insertion (frontal (**C**); oblique (**D**)).

All imaging procedures and 3D analyses were conducted by a single investigator with a high level of experience regarding device handling and software application (Figure 2A–D).

### 2.4. Breast Distance Measurements

Data obtained from the 3D surface scans included the respective pre- and postoperative distances between sternal notch and nipple (Sn-N) and between nipple and inframammary fold (N-M). These pre- and postoperative distances were additionally gauged manually using a tape measure.

### 2.5. Breast Volume Measurements

Using an automatized superimposition algorithm and the volume difference measurement function within the Mirror software, the changes in breast volume were assessed for each stage of the respective surgical procedure. The volumes of the sizers and implants used during surgery were deemed as reference values.

### 2.6. Breast Symmetry Assessment

For each subject, breast symmetry was digitally assessed by comparing the volumes of the left and right breast, as well as by use of a surface deviation analysis. These evaluations were conducted for each pre- and postoperative scan, as well as the respective scans of each implanted sizer.

The previously mentioned surface deviation analysis [23] was performed by superimposing a mirrored 3D image onto the original breast scan: After selecting the breast surface area, an iterative closest point algorithm within the Mirror software was applied to align the reference and mirror images [28]. Subsequently, the root-mean-square error (RMSE) of the point-to-point surface deviation between both breasts was calculated.

A RMSE value of zero was deemed to indicate perfect symmetry, whereas greater values were deemed to represent increasing asymmetry. While there has been an investigation into the correlation of the RSME and the subjective opinion regarding the outcome of breast conserving therapy [26], there were no validated reference values for breast augmentation at the time of conducting this study.

By projecting a heat map onto the 3D surface image, the RSME result was graphically represented within the software. While green coloration indicated areas of lowest surface deviation, blue coloration represented an increase of surface deviation by volume gain, whereas red coloration represented an increase of surface deviation by volume loss.

### 2.7. Statistical Analysis

*p* values below 0.05 were considered to be significantly different. All statistical analyses were performed using IBM SPSS Statistics 25 (IBM, Armonk, NY, USA).

We used Paired two-tailed *t*-tests and Wilcoxon rank-sum tests to examine the differences from the 3D surface scanned distances and manually measured distances in paired continuous data, comparison of 3D volume change to the reference implant volumes, as well as symmetry assessment.

## 3. Results

### 3.1. Subject Demographics

Forty patients were enrolled for this study. The median age was 31 ± 10 (standard deviation) years (range 20–53 years) and mean BMI was 21.8 kg/m² (range 18.1–33.6 kg/m²).

Pre- and postoperative measurements mean that the measurements taken intraoperatively before incision and after suture. A total of 80 pre- and post-operative breasts were evaluated for manual and 3D comparison. Further, 108 cases of breast changes through sizer implantation and exchange were digitally 3D documented.

### 3.2. The Duration of Operation and Scan

The operative time of the study group ranged from 65 to 200 min, with a mean and standard deviation of 94.8 ± 46.7 min. The duration of surgery was less than 3 h in 29 cases and more than 3 h in 11 cases. The scan time was 72 ± 15 s (range 45–100 s).

### 3.3. Comparison of Digital and Manual Breast Distances

Comparison of manual and digital breast distances in the pre- and postoperative state are summarized in Table 1. Both preoperatively and postoperatively comparison in Sn-N and N-M distances showed no significant differences (*p* > 0.05) between manual tape measurement and digital 3D measurement with high correlation. Highest deviations were found between N-M measurements in the postoperative state with a mean (±SD) deviation of 1.23 ± 3.78 mm (range: −11.48 to 8.93 mm)

### 3.4. Comparison of Digital Volume Change and Implant/Sizer Volume

The mean final implant volume was 294.67 ± 75.56 cc (range 125–475 cc). Three-dimensional volume change measured between pre- and postoperative scan after final implant insertion resulted in a mean volume difference of 292.11 ± 76.87 cc. Both measurements showed a high correlation (r = 0.965) and no significant difference between 3D volume changes and implant sizes (*p* = 0.837).

Mean sizer (± SD) volume was 276.85 ± 81.52 cc. Three-dimensional volumetric determined volume for each sizer compared to the preoperative state per breast was 274.38 ± 84.47 cc. Mean volume deviation (± SD) was 0.42 ± 12.31 cc (range: −41.3 to 38.5 cc) There was no statistically significant difference (*p* = 0.860) between breast volume change due to sizer Change and 3D measured volume difference with a high correlation (r = 0.995) (Table 2).

### 3.5. Digital Breast Symmetry Assessment

The volume of the right breast measured by 3D surface scanning had a mean of 189.94 ± 112 cc preoperatively and 479.1 ± 147.1 cc postoperatively. The volume of the left breast showed a mean of 172.65 ± 98.73 cc preoperatively and 471.65 ± 139.88 cc postoperatively. The volume difference between the left and the right breast are neither significant preoperatively (*p* = 0.157), nor postoperatively (*p* = 0.593). The absolute volume difference between the right and the left breast was in average 33.93 ± 30.6 cc preoperatively and 35.66 ± 29.4 cc postoperatively.

Breast symmetry assessed by surface-to-surface deviation was 2.64 ± 1.29 mm RMSE preoperatively and 2.21 ± 1.15 mm RMSE postoperatively. There was no statistically significant difference in breast surface symmetry after breast augmentation (*p* = 0.186) (Table 3).

### 3.6. Surface and Volumetric Changes in Different Sizers

**Figure 3:**
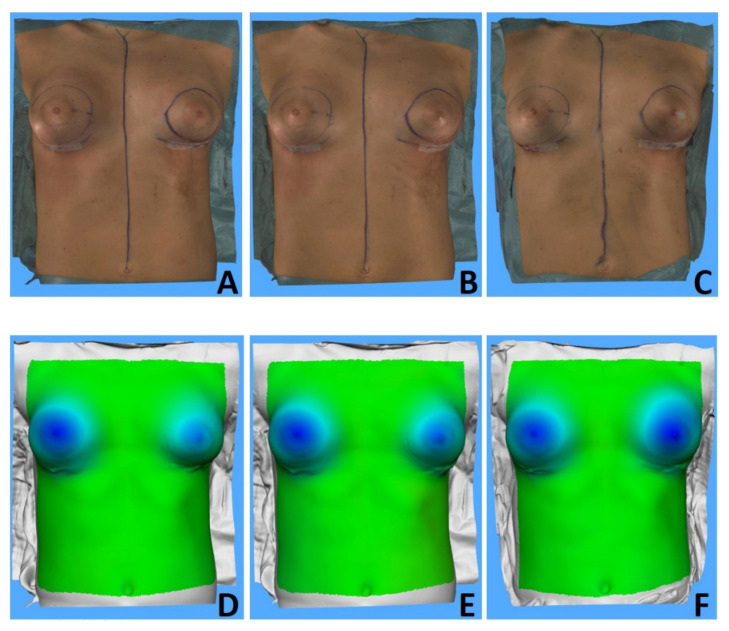
Intraoperative 3DSI frontal views (**A**–**C**) and surface-to-surface analysis (**D**–**F**) of three different sizers placed in the left breast of a 20-year-old patient in comparison to the preoperative image: a 140 cc anatomical low-height moderate-plus sizer (**left row**), a 170 cc anatomical low-height moderate-plus sizer (**middle row**) and a 225 cc round moderate-plus sizer (**right row**). A 195 cc anatomical low-height moderate-plus sizer was placed in the right breast.

**Figure 4:**
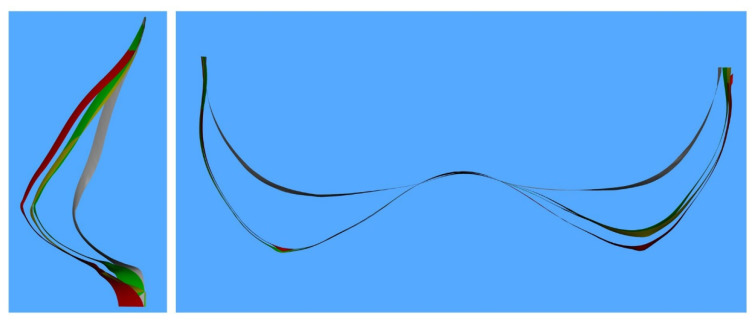
Digital cross-section vertically through the left NAC and horizontally through both NACs. Color-coded pre-operative (**gray**), 140 cc anatomical low-height moderate-plus sizer (**green**), a 170 cc anatomical low-height moderate-plus sizer (**yellow**) and a 225 cc round moderate-plus sizer (**red**). There was no sizer change in the right breast.

A total of 98 sizers were used in the 30 patients ranging from 125 to 475 cc and varied between anatomical and round sizers with different profiles. Comparing 3D changes per breast and individual sizer selection, mean sizer difference (±SD) volume was 92.37 ± 76.87 cc. Three-dimensional volumetric determined volume change between each sizer change per breast was 91.72 ± 75.54 cc. There was no statistically significant difference (*p* = 0.510) between breast volume change due to sizer change and 3D measured volume difference. Figure 3 and Figure 4 showcase different sizer changes (Table 4).

Breast symmetry for implanted sizers was 4.97 ± 4.07 mm RMSE and significantly differed from breast symmetry in postoperative outcome (*p* = 0.003).

## 4. Discussion

In recent years, there have been a growing number of studies evaluating surgical outcomes intraoperatively and postoperatively [29,30,31]. This project was conceived to thoroughly validate 3D mammometrics intraoperatively during breast augmentation in comparison with manual tape measurement and different sizer and implant volumes in 40 patients. We could display that the intraoperative measures before and after surgery were not significantly different when measured using 3DSI or manual measurements. Our findings showed no dramatic differences between the results of intraoperative 3D assessment and manual reference measurements.

Although the use of 3DSI for preoperative consultations and postoperative follow-up in plastic and maxillofacial surgery has become more widespread and intensive over the last few decades, its reliability and usefulness remain controversial [32,33,34,35]. As early as 15 years ago, researchers explored the advantages of using this technique for intraoperative purposes [19,20]. Nevertheless, the application was limited by the extremely high acquisition cost of laser scanners at the time as well as the cumbersome scanning operation and the lack of specialized analysis software. Although the price of 3DSI equipment has now fallen significantly, the intraoperative use of this technology is still not widespread. With this project we hope to demonstrate the potential of intraoperative 3DSI for practical use in the future.

The volume difference between the results calculated using the 3D breast images and the actual implant volume is similar to the expected variance values in previous literature [36,37,38,39]. It can be hypothesized that the implant will generate a volumetric effect on the breast surface. In contrast to studies of 3D breast volume measurements at different timeframes during follow-up, our study focused more on the direct effects after the implantation without distinct hematoma or scarring, and on soft tissue laxity under standardized conditions.

In this study, we used rapid intraoperative assessable 3D measurements, standardized workflows for reproducible validation on uniform patient samples. Before 3DSI can be widely utilized for aid and support in breast augmentation, conclusive framework conditions regarding the accuracy of the analysis results must be confirmed. Compared to published case series in the face [17] and breast region [20], the actual study focuses on a specific and common patient group.

Assessment of breast volume using a wide array of methods as plaster casting, water displacement or MRI have been described in the past [40,41,42]. Apart from measuring the volume to ensure that breasts show symmetry during augmentation, measurement tapes and photographs are widely used to objectify distances pre-, post-, and intraoperatively [43]. Finally, successful breast augmentation relies intraoperatively mainly on the subjective judgement of volume in relation to the body, position of the nipple–areola complex (NAC) and symmetry of the experienced plastic surgeon.

Although the judgement of the aesthetics and the presentation of the post-operative outcomes is largely dependent on the experience and skill of the surgeon, intraoperative 3DSI could take an important role in borderline situations such as significant asymmetrical breasts and the finding of the best matching implant. The use of intraoperative 3DSI may help by giving objectified and precise information about how much volume difference between the breasts is present. Additionally, different projections of the breast can be detected.

Though we did not find a significant difference between the pre- and postoperative surface-symmetry (RMSE value), we could see a decrease in the mean and in the standard deviation. Hence, we could show that postoperatively a more homogenous 3D surface between the left and right breast was achieved compared to the preoperatively. This undermines the significant difference in contrast to the volume percentage. Previous studies showed that 3DSI is an excellent tool for preoperative planning of breast augmentation, albeit while only investigation the predictive value of 3DSI when aiding in the planning process but not during the actual surgery [6,8,38,44,45]. Former studies have shown that the utilization of 3DSI is excellent for monitoring and observing breast changes after augmentation [36,46,47]; however, their analysis and measurements are not beneficial for surgical outcomes after the competition of the procedure. By comparison, the 3D scanner we evaluated in this study can be used in the operating room and thus can directly assist the surgeon in selecting implants of a more appropriate size and shape in order to achieve maximum symmetry. With this method, we can shift the selection procedure from relying solely on the surgeon’s visual control to a more objective process. Although the use of 3DSI makes it easier to determine the volume and shape of implant for optimal symmetry, it is crucial to recognize that at present this approach is still only supplementary to the surgeon’s visual control. Additionally, thorough preoperative communication with the patient to understand his or her needs and expectancy is of the highest priority. This approach requires no adjustments or compromises to the standard surgical environment and the non-contact operation is ideal for intraoperative situation. Moreover, the scan can be finished in less than 30 s and the 3D model just need to be processed in 2 min using the most advanced software before the assessment.

We are aware that our study has several limitations. Study enrollment assessed rather slim patients with small to moderate implant sizes. While this is due to the target client group for breast augmentation surgery, it does facilitate the determination of changes in breast surface and volume. We assume the non-significant difference in digital to manual nipple to inframammary crease to be attributed to the low BMI of the patients. Patients with higher BMI, excessive abdominal and axillary fat pads, high grades of ptosis or preoperative significant higher breast volume will show higher deviations of digital 3D measured surface distances compared to manually taken measurements as 3DSI cannot appreciate the inframammary fold in these cases. This is a known limitation for most 3D scanners and poses an overall limitation to the use of digital breast surface measurements and volume measurements in 3DSI [9,10,37,39,48]. Furthermore, the result of the scan regarding volumetric changes might be dependent on the positioning of the patient, which might change intraoperatively due to movement caused by the surgeon. Another problem with the current use of intraoperative 3DSI is the lack of automated software, which requires a high level of knowledge and confidence in the use of the visualization software.

In addition, we do not have prospective Breast Q or similar patient reported outcome measures on the current study patients. In our future studies, we will collect the subjective patient reported information and pair them with objective measurements.

In addition to breast augmentation with implants, we believe that 3DSI is equally valuable in breast augmentation with autologous fat grafting (AFG). In comparison with implant augmentation, Dr. Gentile found that although the cosmetic results with AFG augmentation were relatively poor after one year, they were more natural, which is more suitable for the treatment of patients with pectus excavatum [49]. The AFG with its gentle technique shows substantially more maintenance than the Coleman procedure [50]. They also found that the patients who received injections of fat combined with adipose-derived stromal vascular fraction cells have better restoration of breast contour and volume through the assessment of MRI, mammography, and ultrasound [51,52]. In our future studies, we will use 3DSI to evaluate and calculate the intra- and post-operative breast contour and volume, so as to provide more comprehensive and objective information on the contrast between breast implant and fat grafting.

In the opinion of the authors, this also includes recently published data on breast height and projection using 3D data [14], the correlation of which can differ greatly both manually and digitally. Time also played a role, as 3D data can be evaluated retrospectively without any problems but should deliver quick results in the intended “live intraoperative setting”. However, based on the data available to us, we are convinced that further digital 3D measurements on the breast surface can be taken automatically and reproducibly in a short time using software.

## 5. Conclusions

There is no difference in the accuracy of intraoperative 3DSI compared to manual measurements, and therefore it can be considered a valid tool. It gives additional detailed analysis data and supports the surgeon in difficult breast augmentation procedures by supplying precise and reliable information on volume variance, which may enable the surgeon to achieve better symmetry. However, limitations of 3DSI may be seen in cases of marked breast hypertrophy and breast ptosis with unclear breast borders. Further studies in the future are needed to validate the method in such cases.

## Figures and Tables

**Table 1:** Comparison of digital and manual breast distances.

Distance	Manual	3D	Delta	Range	*p*	R
Sn-N pre	180.4 ± 14.8	180.8 ± 14.7	−0.4 ± 1.6	−6.3 to 2.2	0.217	0.994
Sn-N post	189.3 ± 16.9	184.8 ± 18.1	0.5 ± 2.00	−4.9 to 5.7	0.120	0.991
N-M pre	64.5 ± 9.9	64.7 ± 9.6	−0.2 ± 1.4	−2.7 to 3.1	0.560	0.990
N-M post	91.1 ± 10.1	89.9 ± 11.2	1.2 ± 3.8	−11.5 to 8.9	0.090	0.939

Table 1 showing the manual and 3D measurements for the sternal notch to nipple distance (Sn-N) and nipple to inframammary fold distance (N-M) at the beginning (pre) and end (post) of surgery, their respective delta and range of deviation with *p*-value and correlation coefficient (R). All measurements are given in millimeters with their respective ± standard deviation.

**Table 2:** Comparison of digital volume change and implant/sizer volume.

Volume	Actual	3D	Delta	Range	*p*	R
Implant	294.67 ± 75.56	292.11 ± 76.87	0.67 ± 9.4	−33.1 to 39.3	0.837	0.965
Sizer	276.85 ± 81.52	274.38 ± 84.47	0.42 ± 12.31	−41.3 to 38.5	0.860	0.995

Table 2 showing the actual volume and 3D volume difference measurements for the implant and sizer at the beginning (pre) and end (post) of surgery, their respective delta and range of deviation with *p*-value and correlation coefficient (R). All measurements are given in cubic centimeter with their respective ± standard deviation.

**Table 3:** Digital breast symmetry assessment.

Volume	Right	Left	Delta	Range	*p*	RMSE
Pre-Op	189.94 ± 112	172.65 ± 98.73	33.93 ± 30.6	−33.1 to 39.3	0.157	2.64 ± 1.29
Post-op	479.10 ± 147.1	471.65 ± 139.88	35.66 ± 29.4	−41.3 to 38.5	0.593	2.16 ± 0.89

Table 3 showing the right and left breast volume and the beginning (pre) and end (post) of surgery, their respective delta and range of deviation with *p*-value and correlation coefficient (R). These measurements are given in cubic centimeter with their respective ± standard deviation. The breast symmetry was assessed by the root-mean-square error (RMSE) of the point-to-point surface deviation between both breasts. It is given in millimeters with their respective ± standard deviation.

**Table 4:** Surface and volumetric changes in different sizers.

Right-Left Breast Difference	Manual/Actual	3D	Delta	Range	*p*	R
Sn-N distance pre	4.3 ± 2.1	3.9 ± 1.8	0.4 ± 0.7	−1.1 to 0.9	0.35	0.992
Sn-N distance post	4.5 ± 1.9	4.2 ± 2.0	0.3 ± 0.5	−1.3 to 0.8	0.41	0.992
N-M distance pre	7.1 ± 1.5	6.6 ± 1.9	1.3 ± 0.6	−1.9 to 1.2	0.47	0.976
N-M distance post	7.4 ± 1.8	7.0 ± 2.1	1.1 ± 0.9	−1.7 to 1.3	0.58	0.989
Sizer Volume	92.37 ± 76.87	91.72 ± 75.54	0.38 ± 2.3	−9.4 to 7.8	0.51	0.985

Table 4 showing the difference of Sn-N and N-M and sizer volume between right breast and left breast at the beginning (pre) and end (post) of surgery, their respective delta and range of deviation with *p*-value and correlation coefficient (R). The distance measurements are given in millimeters with their respective ± standard deviation. The volume measurements are given in cubic centimeter with their respective ± standard deviation.

## Data Availability

Not applicable.
